# The glucose and lipid metabolism reprogramming is grade-dependent in clear cell renal cell carcinoma primary cultures and is targetable to modulate cell viability and proliferation

**DOI:** 10.18632/oncotarget.23056

**Published:** 2017-12-08

**Authors:** Cristina Bianchi, Chiara Meregalli, Silvia Bombelli, Vitalba Di Stefano, Francesco Salerno, Barbara Torsello, Sofia De Marco, Giorgio Bovo, Ingrid Cifola, Eleonora Mangano, Cristina Battaglia, Guido Strada, Giuseppe Lucarelli, Robert H. Weiss, Roberto A. Perego

**Affiliations:** ^1^ School of Medicine and Surgery, University of Milano-Bicocca, Monza, Italy; ^2^ Pathology Unit, ASST Monza, San Gerardo Hospital, Monza, Italy; ^3^ Institute for Biomedical Technologies, National Research Council, Segrate, Italy; ^4^ Department of Medical Biotechnology and Translational Medicine, University of Milano, Segrate, Italy; ^5^ Urology Unit, ASST North Milan, Bassini Hospital, Cinisello Balsamo, Italy; ^6^ Department of Emergency and Organ Transplantation-Urology, University of Bari, Bari, Italy; ^7^ Division of Nephrology, Department of Internal Medicine, School of Medicine, and Cancer Center, University of California, Davis, CA, USA; ^8^ Current address: Pathology Unit, ASST North Milan, Vimercate Hospital, Vimercate, Italy

**Keywords:** renal cell carcinoma, primary cell cultures, glucose and lipid metabolism reprogramming, Fuhrman grade

## Abstract

Clear cell renal cell carcinoma (ccRCC) has a poor prognosis despite novel biological targeted therapies. Tumor aggressiveness and poor survival may correlate with tumor grade at diagnosis and with complex metabolic alterations, also involving glucose and lipid metabolism. However, currently no grade-specific metabolic therapy addresses these alterations. Here we used primary cell cultures from ccRCC of low- and high-grade to investigate the effect on energy state and reduced pyridine nucleotide level, and on viability and proliferation, of specific inhibition of glycolysis with 2-deoxy-D-glucose (2DG), or fatty acid oxidation with Etomoxir. Our primary cultures retained the tissue grade-dependent modulation of lipid and glycogen storage and aerobic glycolysis (Warburg effect). 2DG affected lactate production, energy state and reduced pyridine nucleotide level in high-grade ccRCC cultures, but the energy state only in low-grade. Rather, Etomoxir affected energy state in high-grade and reduced pyridine nucleotide level in low-grade cultures. Energy state and reduced pyridine nucleotide level were evaluated by ATP and reduced 3-(4,5-dimethylthiazol-2-yl)-2,5-diphenyltetrazolium (MTT) dye quantification, respectively. 2DG treatment impaired cell proliferation and viability of low-grade ccRCC and normal cortex cultures, whereas Etomoxir showed a cytostatic and cytotoxic effect only in high-grade ccRCC cultures. Our data indicate that in ccRCC the Warburg effect is a grade-dependent feature, and fatty acid oxidation can be activated for different grade-dependent metabolic needs. A possible grade-dependent metabolic therapeutic approach in ccRCC is also highlighted.

## INTRODUCTION

Clear cell renal cell carcinoma (ccRCC) is the most common (70–80%) and lethal subtype of renal cell carcinomas and accounts for 90% of all kidney cancers [1]. Although biological and targeted therapies have shown promising results for advanced ccRCC [2–3] its prognosis remains poor, with a median overall survival of 21.4 months [4]. In ccRCC the tumor grade at diagnosis may affect survival with a 5-year cancer specific mortality rate ranging from about 7% for grade I to about 58% for grade IV cases [5].

The most striking morphological feature of ccRCC cells is their clear cytoplasm due to lipid and glycogen accumulation [6], suggesting possible involvement of their metabolism in ccRCC progression. Transcriptomic, proteomic and metabolomic profiling of ccRCC tissues support this involvement revealing metabolic reprogramming characterized by up-regulation of aerobic glycolysis (Warburg effect), the pentose phosphate pathway, fatty acid synthesis, glutamine and glutathione metabolism, and by down-regulation of the tricarboxylic acid (TCA) cycle, fatty acid β oxidation (FAO), and oxidative phosphorylation [7–10]. More recently, by using different omics approaches, several groups revealed that specific metabolic alterations, in particular the down-regulation of TCA cycle and the up-regulation of pentose phosphate pathway and fatty acid synthesis, may correlate with tumor aggressiveness and poor survival in ccRCC patients [11, 7]. Hakimi *et al.* investigated the association between metabolic shifts and ccRCC clinical stages, revealing an increase of fatty acid biosynthesis and a decrease of oxidative phosphorylation in early-stage tumors, whereas these metabolic patterns reversed and glutathione metabolism increased in late-stage tumors [8]. A decrease of specific FAO enzyme expression has also been found to correlate with an increase of tumour stage, size and grade, with a concomitant decrease in survival [12]. Furthermore, using a proteomic approach [13], and more recently by combining proteomics and metabolomics analysis [9], it has been revealed that grade-dependent metabolic reprogramming occurs in ccRCC tissues, with the Warburg effect relatively more prominent in higher grade tumors at the expense of the TCA cycle and oxidative metabolism. Grade-dependent alterations were also shown in fatty acid, glutamine and glutathione metabolisms [9]. Even if different targeted therapeutics interfering with various aspects of RCC metabolism are currently in clinical development [14], at present there is no grade-specific therapy addressing this metabolic reprogramming.

To further characterize such grade-dependent reprogramming, in anticipation of rationally developing novel grade-specific metabolic targeted approaches, an *in vitro* model more representative of the ccRCC tissue with respect to the immortalized cell lines, and that maintains grade-dependent tissue metabolic features, would be useful. From ccRCC tissue samples we established primary cell cultures that at the early passages retain the phenotypic, genomic, transcriptomic and proteomic profile of the corresponding tissues, and therefore share many biological processes known to be important for ccRCC development and progression [15–18].

Here we aim to investigate how the specific inhibition of glycolysis or fatty acid oxidation affect the reduced pyridine nucleotide level and energy state and consequently the viability and proliferation of low- and high-grade ccRCC cells. The primary cell cultures from ccRCC of low- and high-grade, which retain the metabolic phenotype of the corresponding tissues, were essential for these purposes.

## RESULTS

### The “clear cell” morphology of ccRCC tissues due to neutral lipid and glycogen storage is maintained in primary cell cultures

**Figure 1:**
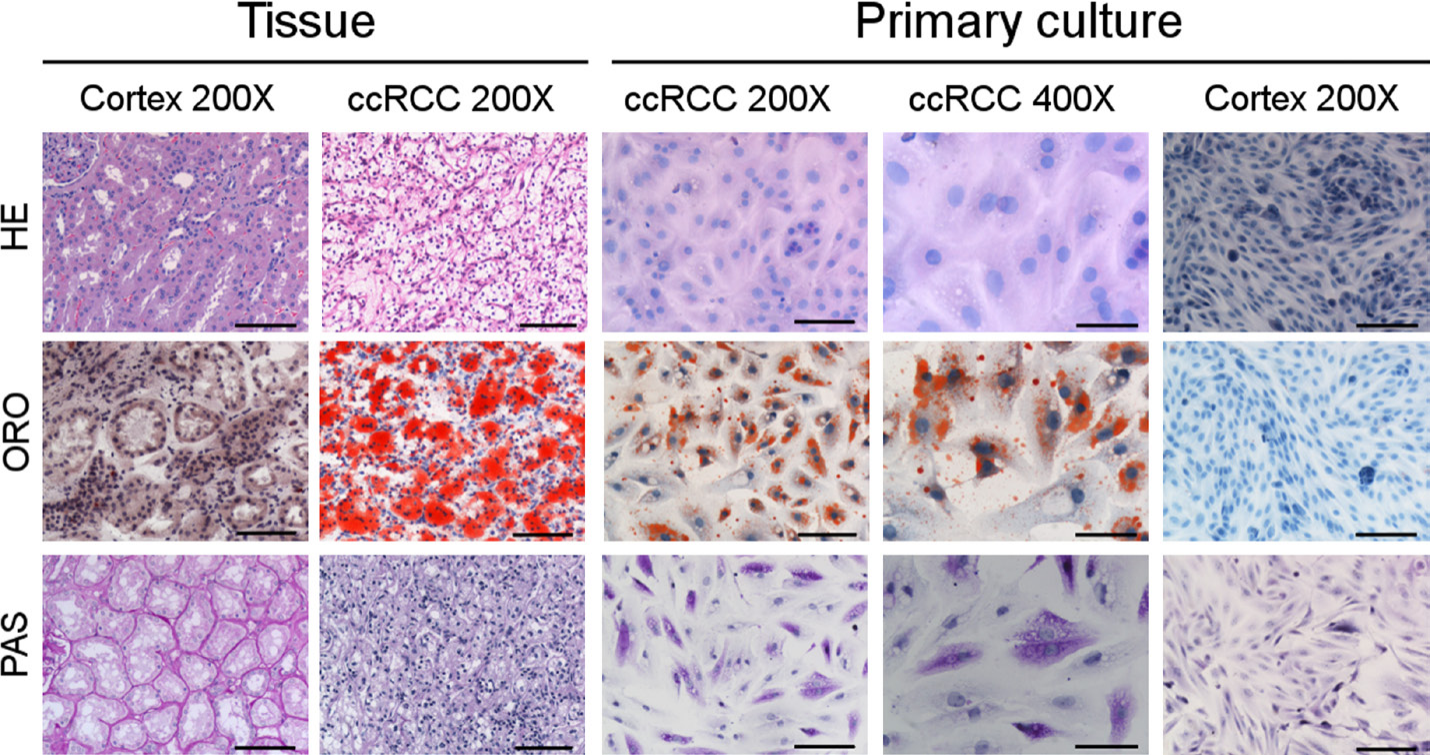
ccRCC primary cell cultures maintain the lipid and glycogen storage of tissues Representative images of normal cortex and ccRCC tissue sections and primary cell cultures after Haematoxylin/Eosin (HE), Oil Red O (ORO) and Periodic Acid-Schiff (PAS) staining captured at original magnification of 200× (scale bar: 100 μm) and 400× (scale bar: 50 μm).

Hematoxylin/Eosin (HE) staining showed that ccRCC primary cell cultures maintained the typical vacuoles responsible for the “clear cell” morphology of corresponding ccRCC tissue. The “clear” vacuoles contained neutral lipids and glycogen, as proved by Oil Red O (ORO) and Periodic Acid-Schiff (PAS) staining respectively, in both ccRCC tissues and primary cell cultures (Figure 1).

### Metabolic gene expression profile of ccRCC primary cultures

**Figure 2:**
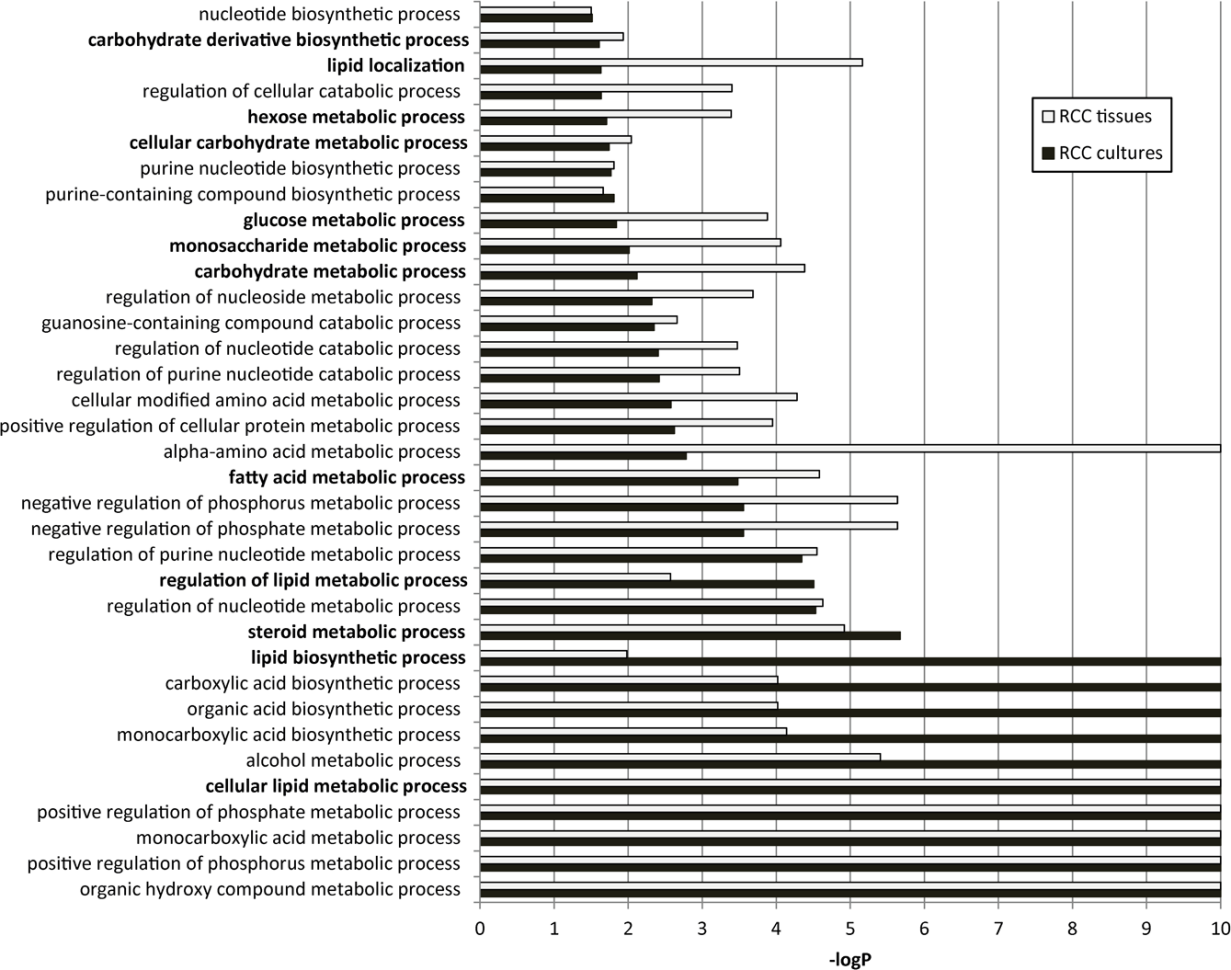
Enrichment for GO biological processes related to metabolism in ccRCC primary cultures and tissues The 35 significant metabolic GO-BP terms shared between cultures (black bars) and tissues (white bars) are represented. The -log *P* value (−log*P*) represents the significance level. The twelve GO-BP terms specifically related to carbohydrate and lipid metabolism are indicated in bold.

To highlight the metabolic signature of ccRCC primary cultures we revised our previously published gene expression microarray data, generated on a collection of ccRCC and normal cortex primary cultures, and corresponding Gene Ontology (GO) enrichment analysis [17] to focus on metabolic processes. In ccRCC primary cultures, we found a significant enrichment for 35 GO-biological process (BP) terms related to several classes of metabolic processes (Figure 2). The same GO enrichment analysis performed on publicly available ccRCC tissue transcriptomic data generated by RNAseq technology [19] evidenced that these 35 metabolic processes were also shared by tumor tissues (Figure 2). In particular, among them we found 12 GO-BP terms specifically related to carbohydrate and lipid metabolism. These metabolic processes were reported as characteristics of ccRCC biology even in other transcriptomic analyses performed on tissues [20].

These findings indicate that ccRCC primary cultures retained, on the whole, the metabolic gene profiling of tumor tissues, in particular the gene profiling of glucose and lipid metabolism, supporting the use of these cultures for accurate metabolic functional studies.

### Lactate fermentation is upregulated in ccRCC primary cell cultures

**Figure 3:**
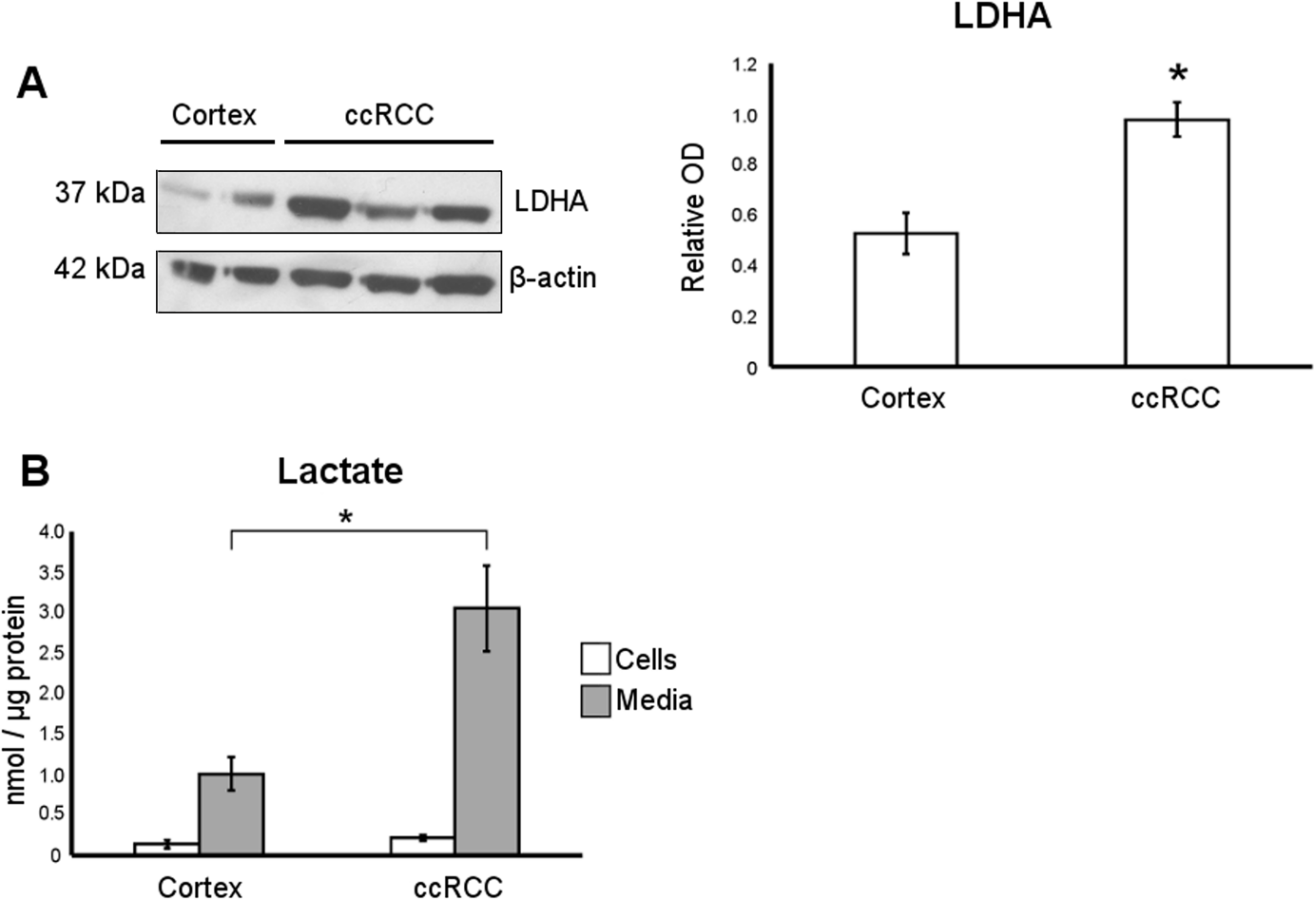
Lactate production is upregulated in ccRCC primary cell cultures **(A)** Representative western blot of normal cortex (*n* = 2) and ccRCC (*n* = 3) primary cell culture, showing LDHA and β-actin proteins. In the graph, the normalized LDHA band intensities of normal cortex (*n* = 7) and ccRCC (*n* = 19) primary cell cultures are shown. (**B)** Lactate quantification performed in cell lysates (cells) and conditioned culture medium (media) of normal cortex (*n* = 6) and ccRCC (*n* = 7) primary cultures. Lactate concentration values were normalized to cell protein concentration. Data expressed as mean ± SEM; ^*^p < 0.05.

To further improve the metabolic characterization of our cultures, we measured lactate dehydrogenase A (LDHA) expression and L-lactate production as probes for aerobic glycolysis/lactate fermentation. An increment of LDHA protein level, the enzyme involved in converting pyruvate to lactate, and both intracellular and secreted L-lactate was observed in our ccRCC cultures as compared with normal cortex cultures (Figure 3A and 3B). These data confirm that the aerobic glycolysis/lactate fermentation was upregulated in our ccRCC primary cell cultures, in agreement with the behavior of ccRCC tissues [9].

### The storage of neutral lipids and glycogen and the production of lactate are grade-dependent in ccRCC primary cell cultures

**Figure 4:**
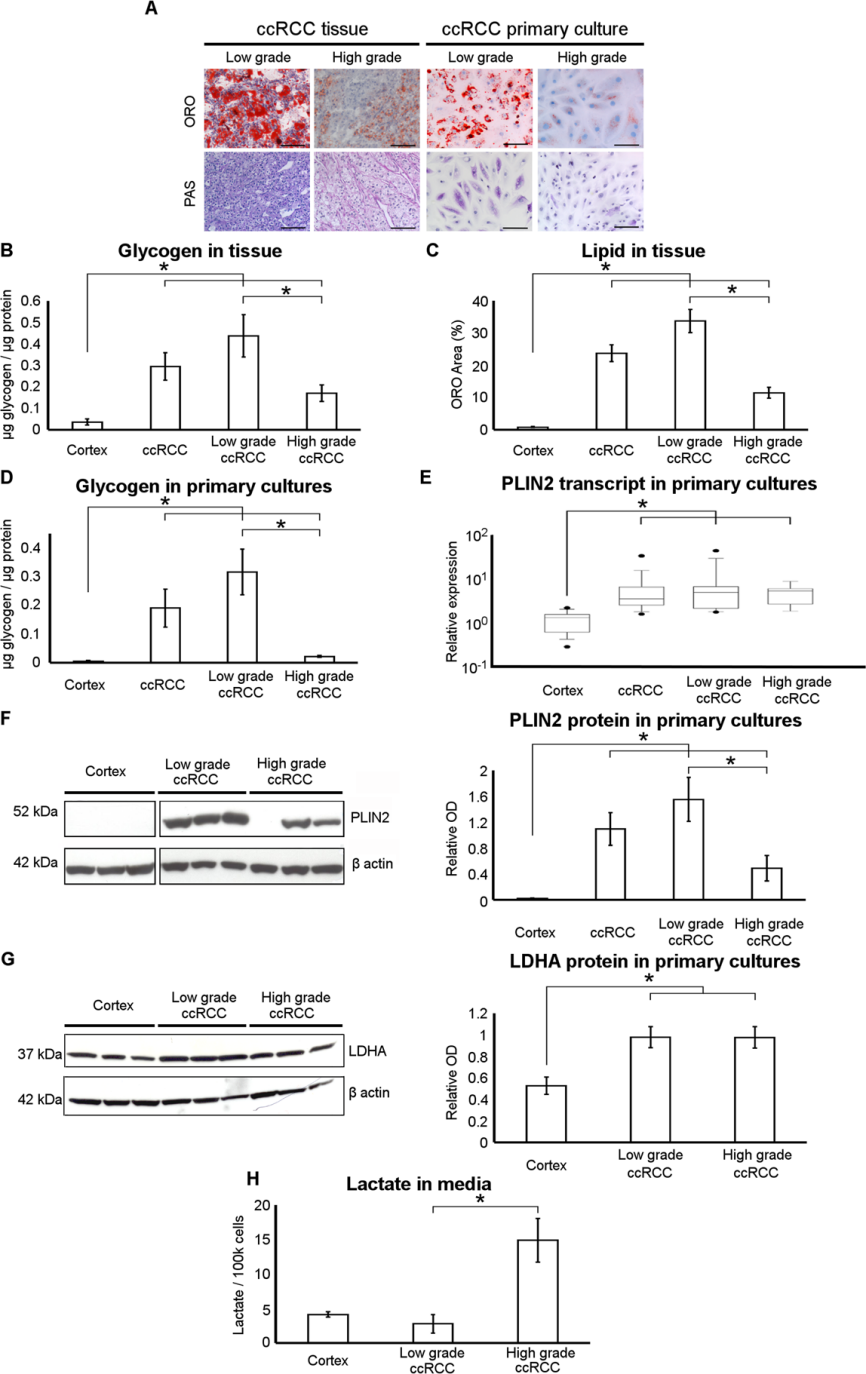
Neutral lipid and glycogen storage is decreased and lactate production increased in high-grade ccRCC primary cultures **(A)** Representative images of low-grade and high-grade ccRCC tissue sections and matched primary cell cultures after Oil Red O (ORO) and Periodic Acid-Schiff (PAS) staining captured at original magnification of 200× (scale bar: 100 μm). (**B)** Glycogen quantification performed in normal cortex (*n* = 4), low-grade (*n* = 7) and high-grade (*n* = 8) ccRCC tissue samples. (**C)** Neutral lipid quantification in ORO stained slides of normal cortex (*n* = 3), low-grade (*n* = 7) and high-grade (*n* = 8) ccRCC tissue samples. ORO area was quantified in three to six fields per slides and expressed as percentage of total area analyzed. (**D)** Glycogen quantification performed in normal cortex (*n* = 3), low-grade (*n* = 4) and high-grade (*n* = 3) ccRCC primary cultures. (**E)** Real-time PCR analysis of PLIN2 expression performed in normal cortex (*n* = 15), low-grade (*n* = 11) and high-grade (*n* = 9) ccRCC primary cultures. Box and whiskers plot corresponds to 1–99^th^ percentiles (bars), 25–75^th^ percentiles (box), and median (line in box). (**F)** Representative western blot of three different normal cortex, low-grade and high-grade ccRCC primary cell cultures showing the PLIN2 and β-actin proteins. The graph shows the normalized PLIN2 band intensities of normal cortex (*n* = 8), low-grade (*n* = 8) and high-grade (*n* = 7) ccRCC primary cultures. To evidence the difference between normal cortex and all ccRCC cultures, in B-F panels the average of low and high-grade ccRCC data is also reported. (**G)** Representative western blot of three different normal cortex, low-grade and high-grade ccRCC primary cell cultures showing the LDHA and β-actin proteins. The graph shows the normalized LDHA band intensities of normal cortex (*n* = 7), low-grade (*n* = 11) and high-grade (*n* = 8) ccRCC primary cultures. (**H)** Lactate quantification performed in conditioned culture medium of normal cortex (*n* = 5), low- grade (*n* = 4) and high-grade (*n* = 3) ccRCC primary cultures. Data expressed as mean ± SEM; ^*^p < 0.05.

Morphological evaluation of the lipid and glycogen storages in ccRCC primary cell cultures and corresponding tissues stratified on the basis of Fuhrman low-grade (G1–G2) and high-grade (G3–G4), showed a decrease of ORO-stained lipid droplets and PAS-stained glycogen content in higher grade ccRCC primary cultures and in the corresponding tissue samples (Figure 4A). These observations were quantitatively confirmed by the evaluation of intracellular glycogen content in tissue samples and primary cell cultures (Figure 4B and 4D) and of ORO stained area in tissue slides (Figure 4C). The grade-dependent decrease of lipid storage was also quantitatively confirmed in primary cell cultures by analyzing the expression of PLIN2, the lipid droplet coat protein of non-adipose tissues described as a marker of intracellular neutral lipid storage [21]. PLIN2 transcript and protein expression was significantly lower in normal cortex with respect to ccRCC primary cell cultures (Figure 4E and 4F), and notably PLIN2 protein level was lower in high-grade compared to low-grade ccRCC cells.

Moreover, to evaluate whether in ccRCC cultures aerobic glycolysis/lactate fermentation was also grade-dependent, we assayed LDHA protein expression and lactate secretion of low-grade and high-grade ccRCC primary cultures. Western blot analysis showed that LDHA protein level was increased in all grade ccRCC cultures as compared with normal cortex cells (Figure 4G), but L-lactate was significantly more abundant in culture media of high-grade with respect to low-grade ccRCC cells (Figure 4H). This discrepancy between LDHA expression and lactate secretion may be due to the fact that the activity of an enzyme may change even without an alteration of its protein concentration, as also previously noted [9]. These data indicate that aerobic glycolysis/lactate fermentation was upregulated in our high-grade ccRCC primary cell cultures as described in high-grade ccRCC tissues [9].

### 2DG treatment decreases the lactate, ATP and reduced MTT dye level in ccRCC cultures of high-grade but the ATP level only in those of low-grade

**Figure 5:**
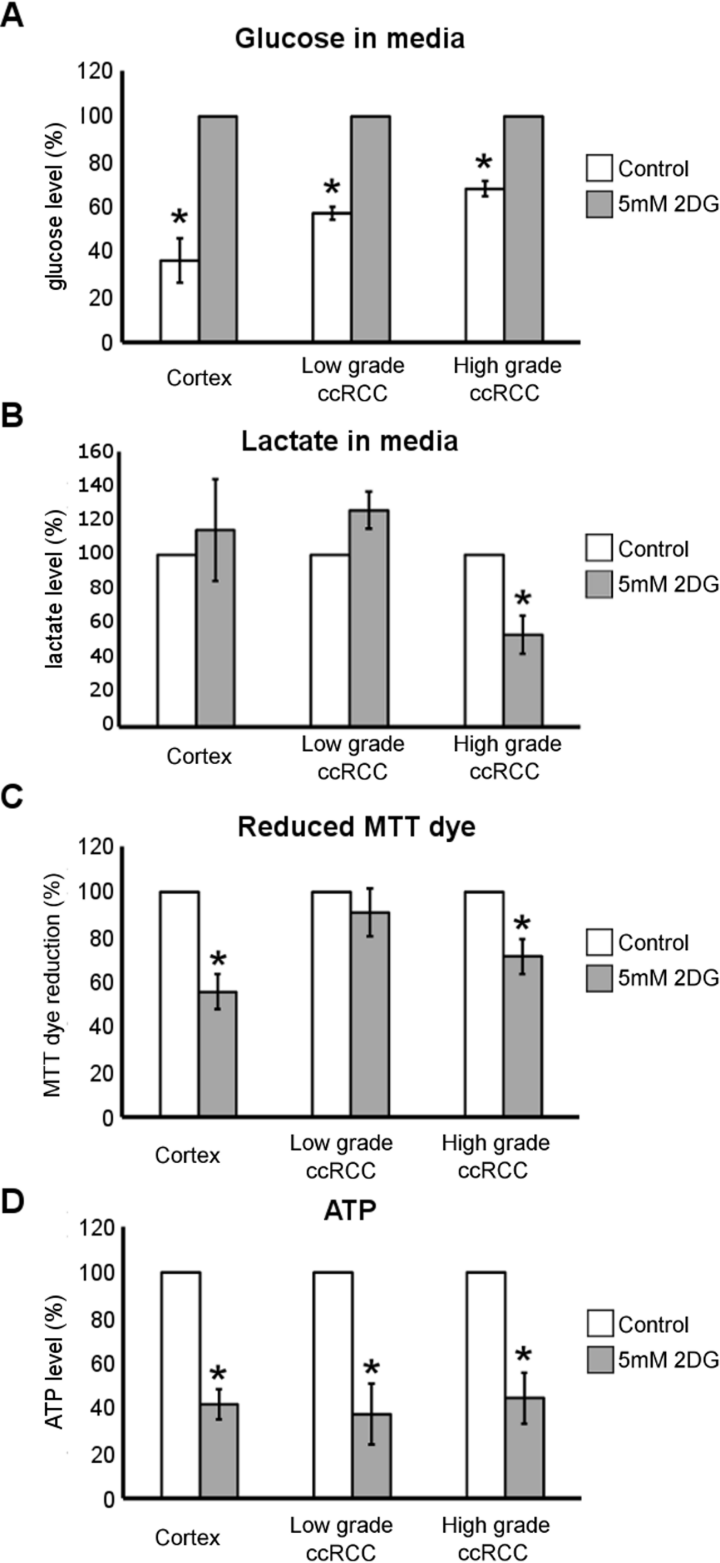
Metabolic effect of 2DG treatment in low- and high-grade ccRCC and normal cortex primary cultures **(A)** Glucose quantification performed in conditioned culture medium of normal cortex (*n* = 3), low- grade (*n* = 4) and high-grade (*n* = 3) ccRCC primary cultures treated for 24 hours with 5 mM 2DG. (**B)** Lactate quantification performed in conditioned culture medium of normal cortex (*n* = 4), low-grade (*n* = 3) and high-grade (*n* = 3) ccRCC primary cultures treated for 24 hours with 5 mM 2DG. (**C)** Quantification of reduced MTT dye performed by MTT assay in normal cortex (*n* = 4), low-grade (*n* = 9) and high-grade (*n* = 5) ccRCC cultures treated for 72 hours with 5 mM 2DG. (**D)** Quantification of ATP content in normal cortex (*n* = 3), low-grade (*n* = 5) and high- grade (*n* = 4) ccRCC cultures treated for 24 hours with 5mM 2DG. All data are represented as percentage with respect to corresponding control (untreated) cells considered equal to 100%, except for panel A in which the treated cells are considered equal to 100%. Data expressed as mean ± SEM; ^*^p < 0.05.

To study whether glucose metabolism contributed to reduced pyridine nucleotide level and energy state of low- and high-grade ccRCC cells, we treated primary cultures with 5 mM 2-Deoxy-D-glucose (2DG). 2DG is known to be phosphorylated by hexokinase in the first step of glycolysis resulting in a non-hydrolysable substrate which accumulates in the cells and leads to inhibition of both glycolysis and pentose phosphate pathway [22]. After 24 hours of treatment the residual glucose level in the medium of normal cortex and ccRCC cultures was significantly higher than in corresponding control medium (Figure 5A) proving that 5 mM 2DG efficiently inhibited the first step of glycolytic pathway. As expected, the 2DG treatment did not induce a decrease of lactate secretion into the medium of normal cortex cultures (Figure 5B), supporting the notion that normal cells do not preferentially use lactate fermentation to metabolize glucose. Even in low-grade ccRCC cells 2DG did not decrease lactate production. Instead, 2DG treatment significantly decreased lactate secretion in high-grade cells (Figure 5B), confirming that the aerobic glycolysis/lactate fermentation is up-regulated in high-grade ccRCC primary cultures, as shown in Figure 4H.

Moreover, treatment with 5 mM 2DG for 72 hours significantly affected the capability of normal cortex and high-grade but not low-grade ccRCC cells to chemically reduce the 3-(4,5-dimethylthiazol-2-yl)-2,5-diphenyltetrazolium (MTT) dye (Figure 5C). The bioreduction of MTT dye is a NAD(P)H-dependent process [23] and thus it can be used as probe for evaluating the changes in reduced pyridine nucleotide level (NADH and to a lesser extent NADPH) [24].

Twenty-four hours of 2DG treatment also significantly affected ATP level in normal cortex and both high- and low-grade ccRCC cells (Figure 5D). Thus, the treatment with 2DG induced a concomitant decrease of lactate, NAD(P)H and ATP level only in high-grade ccRCC cells, and these data highlight that glucose metabolism driven through aerobic glycolysis mainly contributed to reduced pyridine nucleotide level and energy state of these cells.

### Etomoxir treatment decreases the ATP level in ccRCC cultures of high-grade and the reduced MTT dye level in those of low- grade

**Figure 6:**
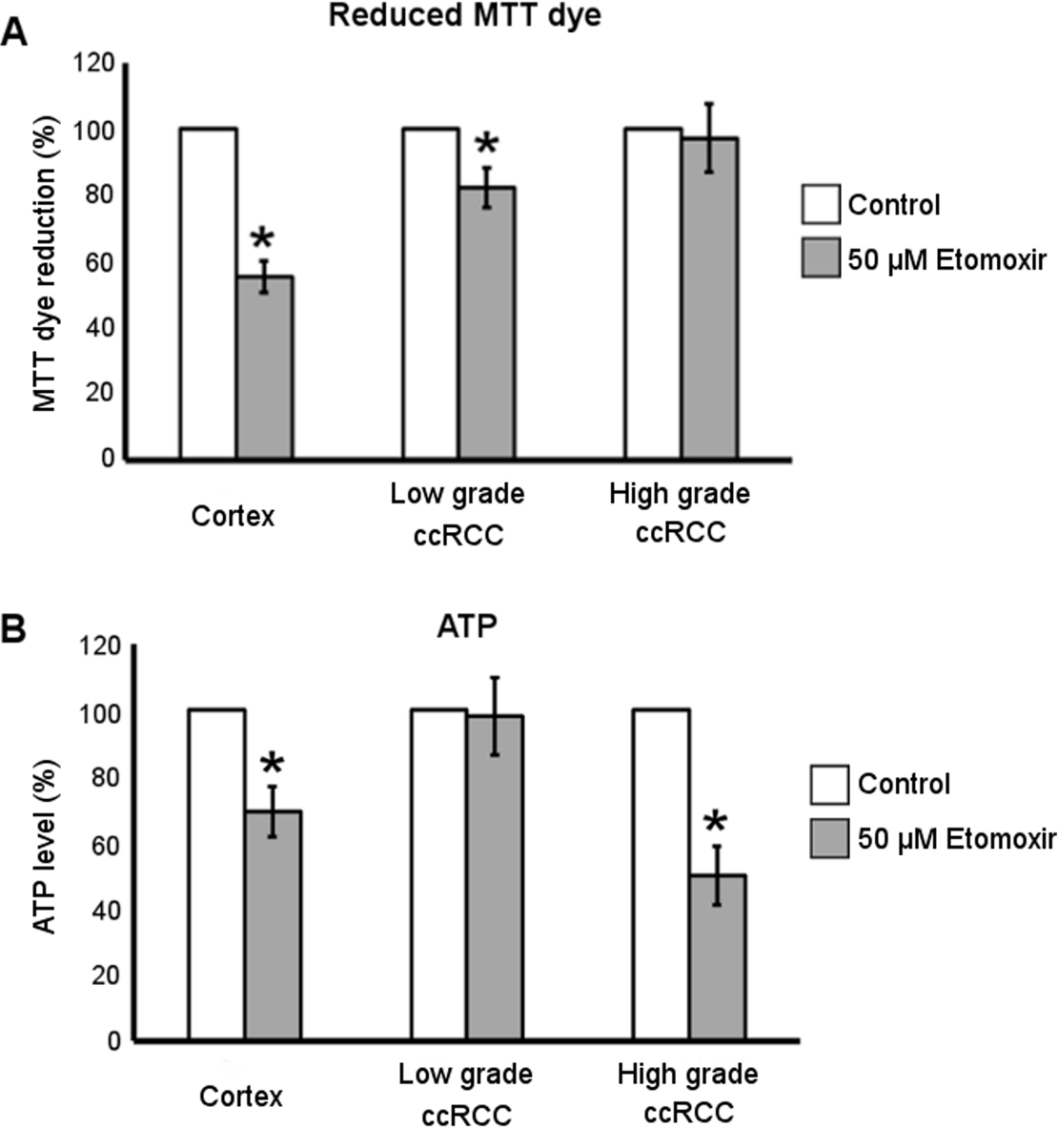
Metabolic effect of Etomoxir treatment in low- and high-grade ccRCC and normal cortex primary cultures **(A)** Quantification of reduced MTT dye performed by MTT assay in normal cortex (*n* = 10), low-grade (*n* = 11) and high-grade (*n* = 13) ccRCC cultures treated for 72 hours with 50 µM Etomoxir. (**B)** Quantification of ATP content in normal cortex (*n* = 3), low-grade (*n* = 4) and high-grade (*n* = 4) ccRCC cultures treated for 24 hours with 50 µM Etomoxir. Data, expressed as mean ± SEM, are represented as percentage with respect to corresponding control (untreated) cells; ^*^p < 0.05.

To evaluate whether lipid metabolism contributed to reduced pyridine nucleotide level and energy state of low-grade and high-grade ccRCC cells, we treated primary cultures with Etomoxir known to inhibit the mitochondrial fatty acid transporter Carnitine palmitoyl transferase 1 (CPT1), responsible for synthesis and import of fatty acylcarnitines across the outer mitochondrial membrane. The reaction catalysed by CPT1 is considered the rate-limiting step of fatty acid β-oxidation [25]. It has been previously shown [9] that the treatment with 50 µM Etomoxir for 72 hours induced an increase of lipid storage in both normal cortex and ccRCC primary cultures, proving that this drug concentration efficiently inhibited CPT1 in our cells. Here we show that the treatment with 50 µM Etomoxir also significantly affected the capability of normal cortex and low-grade but not high-grade ccRCC cells to bioreduce MTT dye (Figure 6A), whereas 24 hours of Etomoxir treatment affected ATP level in normal cortex and high-grade but not in low-grade ccRCC cells (Figure 6B). These data highlight that the inhibition of fatty acid oxidation affected only the reduced pyridine nucleotide level in low-grade and only the energy state in high-grade ccRCC cells, suggesting a different grade-dependent metabolic function of β oxidation process in ccRCC.

### Cell proliferation and viability is affected by 2DG in low-grade ccRCC cultures and by Etomoxir in those of high-grade

**Figure 7:**
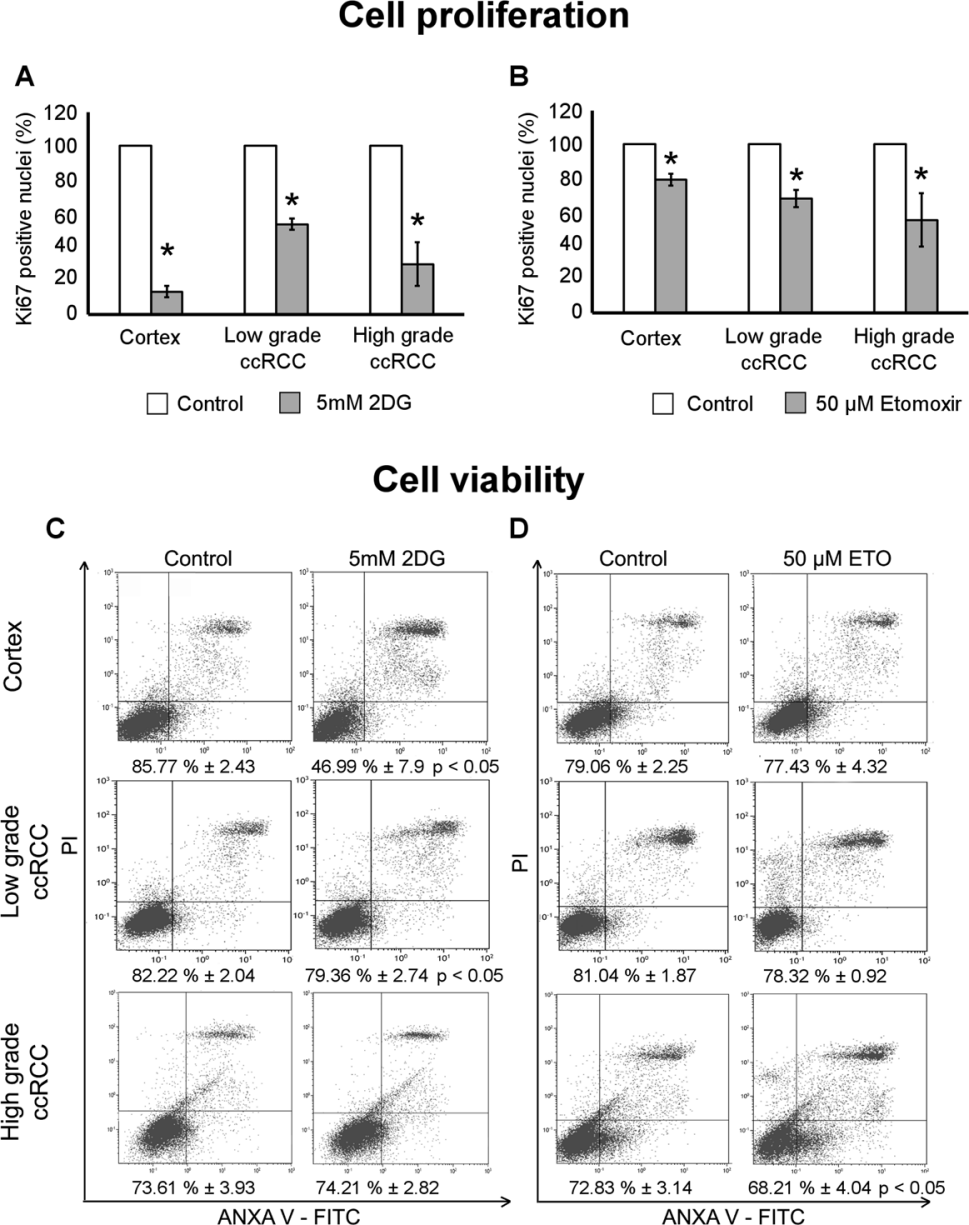
Cell proliferation and viability of low- and high-grade ccRCC and normal cortex primary cultures treated with 2DG or Etomoxir (**A–B**) Quantification of cellular proliferation evaluated as Ki67 positive cells after immunofluorescence staining in normal cortex (*n* = 3), low-grade (*n* = 4) and high-grade (*n* = 5) ccRCC cultures treated for 72 hours with 5 μM 2DG (A) or 50 μM Etomoxir (B). Data, obtained in at least five 400x micrographs per sample, were represented as percentage with respect to corresponding control (untreated) samples; ^*^p < 0.05. (**C–D**) Representative images of FACS analysis of cell viability evaluated with Annexin V/propidium iodide in normal cortex (*n* = 4), low-grade (*n* = 4) and high-grade (*n* = 5) ccRCC cultures treated for 72 hours with 5mM 2DG (C) or 50 μM Etomoxir (D). Percentages of viable cells (bottom left quadrant of each plot) are indicated; ^*^p < 0.05, paired Student’s *t*-test. Data are expressed as mean ± SEM.

To evaluate whether the inhibition of glycolysis or fatty acid oxidation might have a cytostatic effect in our ccRCC cultures, we quantified the cells positive for the proliferation marker Ki67 after 72 hours of treatment with 2DG or Etomoxir. Both treatments significantly decreased cell proliferation in normal cortex and in low- and high-grade ccRCC cultures (Figures 7A and 7B).

Instead, the cell viability, evaluated by FACS after Annexin V/IP staining, was differently affected based on the metabolic treatment. 2DG induced a significant decrease of cell viability in normal cortex and low-grade ccRCC cultures (Figure 7C). In normal cortex cultures the viability dropped to 47% and the apoptotic cells raised from about 12% to 51%, in low-grade ccRCC cultures the viable cells decreased to 79% and the apoptotic cells raised from 15% to 19%. Etomoxir induced a significant decrease of viable cells in high-grade ccRCC cultures only, the viable cells decreased to 68% and the dead cells significantly raised from 19% to 23%. It is noteworthy that the inhibition of fatty acid oxidation did not affect the viability and just slightly the proliferation of normal cortex cells, whereas the inhibition of glycolysis severely affected both their viability and proliferation.

These data evidence that a cytostatic and cytotoxic effect can be obtained by inhibition of glycolysis in low-grade ccRCC cells and by inhibition of fatty acid oxidation in high-grade ccRCC cells. The extension of treatment until five days did not quantitatively increase the difference in viability between control and treated cells (data not shown).

## DISCUSSION

In this study we showed that ccRCC primary cell cultures maintained in early passages the cytological, transcriptomic and metabolic features of the original tumor tissues, confirming the up-regulation of aerobic glycolysis/lactate fermentation. Our primary cultures also showed a grade-dependent modulation of lipid and glycogen storage and aerobic glycolysis/lactate fermentation, accordingly to the grade-dependent metabolic reprogramming described in ccRCC tissues [9], and highlighted their suitability as ccRCC *in vitro* model for metabolic studies. The metabolic treatments of these ccRCC primary cell cultures induced interesting modifications in their energy state, reduced pyridine nucleotide level, cell proliferation and viability.

The treatment with 2DG significantly affected the capability of high-grade ccRCC cells, but not low-grade, to chemically reduce the MTT dye. MTT assay was here used as probe for the evaluation of changes in cellular reduced pyridine nucleotide level, NADH and to a lesser extent NADPH [23–24], to which the mitochondrial TCA and β-oxidation pathways, but also glycolysis and the pentose phosphate pathway, contribute. In 2DG treated high-grade ccRCC cells the decrease of reduced MTT dye level, associated with the decrease of lactate secretion, suggests involvement of glucose metabolism driven through the aerobic glycolysis/lactate fermentation pathway in NAD(P)H production. In fact, in high-grade cells fatty acid oxidation did not appear relevant for NAD(P)H production because Etomoxir treatment did not significantly affect the cell capability to reduce MTT dye. Thus, in our high-grade ccRCC cultures the glucose metabolic pathway had a peculiar role in NAD(P)H production, although not fuelled by the poor glycogen storage.

In low-grade ccRCC cells the poor production of lactate in spite of glucose consumption, and the unchanged lactate secretion after 2DG treatment, demonstrated that glucose underwent mitochondrial catabolism in these cells. Otherwise, in our low-grade ccRCC cells the use of mitochondrial metabolism was also evidenced by the significant decrease of reduced MTT dye after the treatment with Etomoxir that inhibits mitochondrial fatty acid metabolism. However, the fact that MTT data did not change after treatment of these cells with 2DG suggested that the mitochondrial metabolism of glucose contributed only marginally to the NAD(P)H production in low grade ccRCC cells.

2DG treatment in low-grade ccRCC cells significantly affected ATP production that instead was not affected by the inhibition of fatty acid oxidation with Etomoxir. This result together with the unchanged lactate secretion after 2DG treatment highlighted the importance of the mitochondrial metabolism of glucose for energy production that in these low-grade cells might be also fuelled by the large glycogen storage. In high-grade ccRCC cells ATP production was significantly affected by Etomoxir, other than 2DG, suggesting that these cells relied mainly on fatty acid oxidation, other than on aerobic glycolysis/lactate fermentation, for ATP production. In fact, the decrease of ATP in high-grade cells, in presence of 2DG, evidenced that the poor glycogen storage was not enough for efficiently fuelling the aerobic glycolysis that resulted to be inadequate to sustain alone the energy balance in these cells. As expected, the treatment with 2-DG did not affect lactate production in normal cortex cultures but, like Etomoxir, induced a decrease of reduced MTT dye and ATP level. This result confirmed the well-known relevant role that both glucose and lipid metabolism have in normal cells for NAD(P)H production and ATP synthesis, through mitochondrial oxidative phosphorylation.

In our *in vitro* model the inhibition of glycolysis with 2DG affected the viability of normal cortex and low-grade ccRCC cells. Conversely, the inhibition of fatty acid oxidation by Etomoxir induced a significant decrease of viable cells in high-grade ccRCC cultures only, without affecting cortex cell viability. These cytotoxic effects might be due to the significant decrease of ATP production mainly obtained by fatty acid oxidation in high-grade ccRCC cells and by mitochondrial glucose metabolism in low-grade cells.

Even the decreased cell proliferation observed after 2DG or Etomoxir treatment might be due to the decrement of ATP. In particular, the cytostatic effect of Etomoxir agrees with data showing a cell cycle arrest in Caki-1 and 786-O ccRCC cell lines treated with Peroxisome proliferator-activated receptor a (PPARa) antagonists [26] that, downregulating the PPARa target CPT1 [27], might in part reproduce the metabolic and functional effects of Etomoxir. In low-grade ccRCC cells the cytostatic effect of Etomoxir seems to correlate with the observed decrease of the NAD(P)H-dependent MTT dye reduction. In cancer cells it is well known that fatty acid oxidation is relevant, through metabolic pathways fuelled by cytosolic citrate, to the production of NADPH involved in the synthesis of membrane fatty acids during cell proliferation [28]. The decrease of reduced MTT dye level, observed in 2DG treated high-grade ccRCC cells, might in part justify, through the same mechanism, the cytostatic effect in these cells.

Therefore, our data evidenced that in high-grade ccRCC cells fatty acid oxidation mainly, but also aerobic glycolysis, are important for energy production, whereas glucose metabolism uniquely contributes to the reduced pyridine nucleotide level. Instead, in low-grade ccRCC cells the fatty acid oxidation appeared to control the reduced pyridine nucleotide level, while the mitochondrial glucose metabolism controlled the cell energy state.

It is of note that the prevalent aerobic glycolysis (Warburg effect) and the active β-oxidation of our high-grade ccRCC cells have a strict link with the nuclear morphological features of high-grade Fuhrman classification, which are characteristic of cycling or immature cells [29] and correlate with the more aggressive behavior of high-grade ccRCC tumor that has the worst prognosis [5]. The Fuhrman grade and the cell proliferation/cycling characteristics correlate even in our primary cell cultures in which a larger percentage of Ki-67 positive cells was measured in high-grade with respect to low-grade ccRCC cultures (33% versus 18%). In addition, the metabolic characteristics of our high-grade ccRCC cells justify the described higher 18F-fluorodeoxyglucose uptake of high-grade ccRCC [30] with respect to the low-grade tumors that likely relay on a prevalent oxidative mitochondrial metabolism as the normal cortex cells.

Our findings indicate that the Warburg effect is not a common feature shared by all ccRCC, and the β-oxidation can be activated for different metabolic needs in ccRCC of different grade. Our data also support the current trend that underline the importance of the classification of malignancies even on the basis of metabolic pathway alterations [31]. This trend will promote the use of metabolism reprogramming to open novel opportunities for new diagnostic and therapeutic options [32]. The cytotoxic effect that we observed exclusively in high-grade ccRCC cells and not in normal cortex cells after the inhibition of fatty acid oxidation, can be a strong incentive to attempt a metabolic grade-dependent therapeutic approach, not investigated yet in ccRCC [14].

## MATERIALS AND METHODS

### Tissues

Tumor (*n* = 56) and normal kidney (*n* = 36) tissues were obtained from 56 patients following nephrectomy due to the presence of ccRCC. The tissues collected were those exceeding the diagnostic needs. The normal cortex was taken from a healthy region of the kidney, without any indication of cancer. All procedures were performed after written consent and were approved by the Local Ethical Committee.

The clinico-pathological characteristics of enrolled ccRCC patients, reported in Table 1, also included the pathological stage and Fuhrman grade, according to 2004 World Health Organization classification.

**Table 1:** Clinicopathological variables of 56 ccRCC cases analyzed in the study

Variable	*n* (%)
**Age (years)**	
median	71
range	41–86
**Gender**	
male	37 (66.1)
female	19 (33.9)
**Tumor size (cm)**	
median	4.5
range	2–10
**Pathological stage**	
pT1a	13 (23.2)
pT1b	12 (21.4)
pT2	10 (17.9)
pT3a	21 (37.5)
pN+	1 (1.8)
cM+	3 (5.4)
**Fuhrman grade**	
G1–2	32 (57.1)
G3–4	24 (42.9)

### Primary cell cultures

Primary cell cultures were obtained from normal cortex and tumor tissues, the culture conditions and immunophenotypic characterization were performed as described [15]. The primary cell cultures were used at the first confluence.

### Tissue and primary cell culture staining

HE and PAS staining were conducted on formalin fixed and paraffin-embedded tissue sections, following standard protocols [33], and on first-confluent primary cell cultures grown on glass coverslips and fixed in 10% formalin for 1 hour. ORO staining was performed on cryostat tissue sections and on formalin fixed cultures, as described [9]. The stained samples were analyzed by Nikon Eclipse E800 microscope with 10×, 20× and 40× objectives (Nikon Instruments spa, Firenze, Italy). Three/four pictures for each slide were randomly captured and analyzed by LuciaG Image analysis system (Nikon). Lipid storage quantification was obtained by analysis, with ImageJ software (NIH, Bethesda, MD), of ORO stained tissue pictures captured at 100× magnification.

### Gene expression microarray profiling

ccRCC and normal cortex primary cultures were characterized for transcriptome profile by microarray technology on Affymetrix GeneChip Human Exon 1.0 ST Arrays (Affymetrix, Santa Clara, CA, USA), as we previously described [17]. CEL files are available at Array Express repository under accession number E-MTAB-4074 (http://www.ebi.ac.uk/arrayexpress/experiments/E-MTAB-4074/). The differentially expressed genes (DEGs) between ccRCC and cortex cultures were calculated using Partek Genomic Suite software (Partek Inc., St. Louis, MO) by ANOVA method. Gene Ontology biological processes (GO-BP) significantly enriched in our ccRCC cultures were identified by ToppGene suite (https://toppgene.cchmc.org/). ToppCluster tool (https://toppcluster.cchmc.org/) was used to compare the GO-BP enriched in our ccRCC cultures to those enriched in ccRCC tissues obtained from the RNA-seq DEG list reported by Wozniak *et al.* [19].

### RNA extraction and real-time PCR

Total RNA extraction and reverse transcription (RT) were carried out as previously described [34]. Real-time PCR was performed with TaqMan Gene Expression Assay kits for PLIN2 transcript (Hs 00605340_m1) and for GAPDH (Hs99998805_m1 kit) according to manufacturer’s instructions (Applied Biosystems, Foster City, CA). The amplifications were carried out in 20 μl reactions containing 100 ng of cDNA, 1X Universal PCR Master Mix, and corresponding primers and probes, in duplicate for each sample on an ABI PRISM^®^ 7900HT Fast Real-Time PCR System (Applied Byosystems). PLIN2 transcript levels were represented as relative expression (2^−∆∆CT^) with respect to normal cortex samples.

### Protein extraction and western blot analysis

Thirty μg of protein lysates obtained from first-confluent primary cell cultures, quantified with BCA microassay (Sigma Aldrich, St. Louis, MO) and separated on NuPage 4 to 12% Bis-Tris pre-cast gels (Thermo Fisher, Waltham, MA) [35], were submitted to western blotting [36] with mouse monoclonal antibody against PLIN2 (dilution 1:500; AP125, Progen, Heidelberg, Germany), or rabbit polyclonal antibody against LDHA (dilution 1:1000; Cell Signaling, Boston, MA), or against β-actin (dilution 1:1000; Sigma-Aldrich). Densitometric analysis of specific bands was performed by ImageJ software, and the specific band intensities were normalized with corresponding β-actin for quantification.

### Glycogen quantification

Intracellular glycogen content was quantified in tissue homogenates and first-confluent primary cell culture lysates using a Glycogen Assay kit (Biovision, Milpitas, CA) following the manufacturer’s instructions. Data were expressed as μg per μg of total protein content.

### Metabolic treatments

An equal number of normal cortex and ccRCC cells from first-confluent primary cultures were plated and grown for 24 hours in culture medium (complete DMEM medium containing 5 mM glucose). The cells were then washed with PBS and incubated for 24 or 72 hours in culture medium with 5 mM 2-Deoxy-D-glucose 2DG, or 50 µM Etomoxir (Sigma-Aldrich).

### Lactate quantification

Untreated first-confluent cells were lysed in 50 μl H_2_O and centrifuged to remove the insoluble debris. Twenty μl of the cellular soluble fraction lysate, and of conditioned culture medium of untreated or 24 hour 2DG treated cells, were respectively diluted in 1 ml pre-filled reaction cup containing Glucose/Lactate hemolyzing solution (Biosen, EFK diagnostics, Cardiff, England) and assayed for L-lactate content by a lactate analyzer (BiosenC-Line, EKF diagnostics). Concentration values were normalized for viable cell count or, when indicated, for cell protein concentration.

### Glucose quantification

Five hundred μl of conditioned medium of untreated or 24 hour 2DG treated cells were collected, spinned for 5 min to eliminate floating cells and debris, and assayed for glucose content using Roche COBAS 8000 analyzer (Roche diagnostics spa, Monza, Italy). Values were normalized for viable cell count and expressed as percentage with respect to 2DG treated cells.

### MTT assay

Quantification of the NAD(P)H-dependent capability of cells to reduce MTT dye in presence or absence of metabolic treatment was measured by MTT assay (Sigma-Aldrich) [37]. Briefly, 1 × 10^4^ cells were plated in 96-well plates, and after 72 hour of 2DG or Etomoxir treatment the cells were incubated in MTT solution. After 3 hours, the MTT solution was removed, and the blue crystalline precipitate in each well was dissolved in DMSO. Absorbance of each well at 570 nm was quantified using the microplate reader Victor Wolla C1420 (Perkin Elmer, Woltham, MA) and expressed as percentage with respect to untreated cells.

### ATP quantification

Quantification of cellular ATP content in presence or absence of metabolic treatment was measured using ATP Bioluminescence Assay Kit CLS II (Roche Diagnostics, Mannheim Germany). 1.5 × 10^5^ cells were plated in 12-well plates and treated for 24 hours with 2DG or Etomoxir. ATP content was quantified using the microplate luminometer Victor Wolla C1420 (Perkin Elmer) according to manufacturer’s instructions. Data normalized for cell protein concentration were expressed as percentage with respect to untreated cells.

### Ki67 immunofluorescence staining

To assess the effect of metabolic treatments on cell proliferation of ccRCC and normal cortex primary cultures 1 × 10^5^ cells seeded onto coverslips were treated with 2DG or Etomoxir for 72 hours. Immunofluorescence staining was performed using mouse monoclonal antibody against Ki67 (1:75 dilution; Clone MIB-1; DAKO, Glostrup, Denmark) and Alexa Fluor 488 goat anti-mouse polyclonal secondary antibody (1:100 dilution; Molecular Probes, Carlsberg, CA) as previously described [15]. Nuclei were counterstained with DAPI. For each treatment, Ki67 positive nuclei were quantified in at least five immunofluorescence micrographs, obtained at 400x magnification using a Zeiss LSM710 confocal microscope and Zen2009 software (Zeiss, Oberkochen, Germany), and normalized by total nuclei. Normalized Ki67 positive cells in treated samples were expressed as percentage of corresponding untreated samples.

### Cell viability assay

Cell viability of metabolically treated ccRCC and normal cortex primary cultures was assessed. 2.5 × 10^5^ cells were seeded in 6-well plates and treated with 2DG or Etomoxir for 72 hours. Cell viability was evaluated with FITC Annexin V Apoptosis detection kit and Propidium Iodide (PI) (Biolegend, San Diego, CA) according to the manufacturer’s instructions. Briefly, after two washes with cold PBS, the cells were resuspended in 100 µl of Annexin V Binding Buffer. The cell solution was then incubated with 5 µl of FITC Annexin V and 10 µl of PI for 15 minutes at room temperature in the dark. After the incubation, 200 µl of Annexin V Binding Buffer were added. FACS analysis was performed on 10000 events with MoFlo Astrios Cell Sorter and Kaluza software (Beckman Coulter srl, Milano, Italy). Viable (Annexin V and PI negative), dead (PI positive) and apoptotic (Annexin V positive) cells in treated and untreated samples were expressed as percentage with respect to total number of analysed events.

### Statistical analysis

Data were analysed using unpaired Student’s *t*-test or, where indicated, by paired Student’s *t*-test. The results were expressed as mean ± SEM. *P*-values < 0.05 were considered as statistically significant.
